# Chances for Longevity

**Published:** 1873-04

**Authors:** 


### CHANCES FOR LONGEVITY.

Whether feeble or deformed, or scrawny or fat, thick or thin, long or short, that man has the best chance for reaching three score and ten who eats at three regular times a day, and nothing between ; whose bodily functions are in regular daily action, and who is kept fully employed in agreeable and profitable activities in the open air. At the same time, the lean have advantages over the fat ; the merry over the morose ; the hopeful over the despondent ; the busy over the idle ; the good over the bad, for the wicked "is driven away in his wickedness."

